# Comorbidities between tuberculosis and common mental disorders: a scoping review of epidemiological patterns and person-centred care interventions from low-to-middle income and BRICS countries

**DOI:** 10.1186/s40249-019-0619-4

**Published:** 2020-01-15

**Authors:** André Janse Van Rensburg, Audry Dube, Robyn Curran, Fentie Ambaw, Jamie Murdoch, Max Bachmann, Inge Petersen, Lara Fairall

**Affiliations:** 1 Centre for Rural Health, School of Nursing and Public Health, University of KwaZulu-Natal Howard College, Berea, Durban, South Africa; 2 Knowledge Translation Unit, University of Cape Town Lung Institute, George Street, Mowbray, Cape Town South Africa; 3 College of Medicine and Health Sciences, Bahir Dar University, Bahir Dar, Ethiopia; 4 University of East Anglia School of Health Sciences, Norwich Research Park, Norwich, Norfolk UK; 5 King’s Global Health Institute, King’s College London, Stamford Street, London, UK

**Keywords:** Tuberculosis, Mental disorder, Comorbidity, Person-centred care, Low-to-middle income countries, BRICS

## Abstract

**Background:**

There is increasing evidence that the substantial global burden of disease for tuberculosis unfolds in concert with dimensions of common mental disorders. Person-centred care holds much promise to ameliorate these comorbidities in low-to-middle income countries (LMICs) and emerging economies. Towards this end, this paper aims to review 1) the nature and extent of tuberculosis and common mental disorder comorbidity and 2) person-centred tuberculosis care in low-to-middle income countries and emerging economies.

**Main text:**

A scoping review of 100 articles was conducted of English-language studies published from 2000 to 2019 in peer-reviewed and grey literature, using established guidelines, for each of the study objectives. Four broad tuberculosis/mental disorder comorbidities were described in the literature, namely alcohol use and tuberculosis, depression and tuberculosis, anxiety and tuberculosis, and general mental health and tuberculosis. Rates of comorbidity varied widely across countries for depression, anxiety, alcohol use and general mental health. Alcohol use and tuberculosis were significantly related, especially in the context of poverty. The initial tuberculosis diagnostic episode had substantial socio-psychological effects on service users. While men tended to report higher rates of alcohol use and treatment default, women in general had worse mental health outcomes. Older age and a history of mental illness were also associated with pronounced tuberculosis and mental disorder comorbidity. Person-centred tuberculosis care interventions were almost absent, with only one study from Nepal identified.

**Conclusions:**

There is an emerging body of evidence describing the nature and extent of tuberculosis and mental disorders comorbidity in low-to-middle income countries. Despite the potential of person-centred interventions, evidence is limited. This review highlights a pronounced need to address psychosocial comorbidities with tuberculosis in LMICs, where models of person-centred tuberculosis care in routine care platforms may yield promising outcomes.

## Background

Tuberculosis (TB) remains a significant cause of the global disease burden, especially among disadvantaged populations. In 2018, approximately 10 million people developed clinical disease, of whom about half a million new cases were rifampicin resistant. 1.3 million people co-infected with HIV and 300 000 without HIV died [1]. The inextricable relationship between TB and mental disorders is well known although less well-researched. Mental illnesses such as anxiety, mood and addiction disorders may have a high comorbidity rate among people suffering from TB [2–4], possibly with multidirectional relationships. Mental and substance use disorders increases risk factors to TB disease progression, such as tobacco smoking and poor nutrition, often compounded by additional conditions such as diabetes and HIV infection [2].

Common mental disorders (CMD), including substance abuse, are key mechanisms in medication adherence behaviour [5]. Alcohol use raises the risk of transmission due to patterns of social behaviour (i.e. increased exposure in congregate settings like bars), impairing the immune system and, raising the odds for infection [6]. Stigma in relation to mental illness and TB creates significant barriers to the prevention and cure of TB in many communities. This includes the composite of stigmatising effects of comorbidity between TB, mental disorders and stigmatised diseases such as HIV [7]. In short, both proximal and distal associations between TB and CMDs unfold within multiple biological and psychosocial causal pathways, even though these mechanisms remain under-researched [3]. The bulk of TB diseases is experience in low-to-middle income countries and emerging economies (BRICS: Brazil, Russia, India, China and South Africa) [1]. However, there limited research that suggests that treatment of psychological aspects of TB may lead to improved clinical outcomes.

In India, psychotherapy during treatment resulted in improved adherence, treatment and cure outcomes [8]; group-based psychological support for patients with multidrug resistant tuberculosis (MDR-TB) in Peru improved treatment outcomes [9]; and in Ethiopia, ‘TB clubs’ both reduced stigma and improved treatment outcomes among patients [10], and an educational and psychological intervention improved treatment adherence [11].

The need to comprehend and address the TB-mental illness nexus has become more pronounced in low-to-middle income countries (LMICs) against the drive towards more holistic, intersectoral, person-centred health systems [12], backed by the Sustainable Development Goals (SDGs) [13]. Following these directives, there is a pressing need to develop and refine evidence-based interventions towards improving person-centred care in addressing TB and common mental disorder comorbidity. There is however a distinct gap in current literature that systematically describes and examines the LMIC epidemiology of TB and mental disorders, as well as best practices in addressing this complex comorbid condition in LMIC health systems. The present study is located within a larger research consortium brought together to address key challenges plaguing health systems in sub-Sahara African countries (ASSET: heAlth System StrEngThening in sub-Saharan Africa), funded by the National Institute for Health Research (NIHR) in the United Kingdom [14]. One of the research packages of this programme focuses on developing person-centred care (PCC), especially in the contexts of comorbidities between TB and mental disorders.

PCC is an important ideal for TB treatment and care, a disease with a long and damaging social history, the diagnosis of which causes profound stigma, marginalisation and harm to the sense of self and personhood [15] – especially in conjunction with mental disorders [3]. Person-centred interventions have yielded significant benefits in improving psychological well-being and positive effects on behavioural symptoms and treatment adherence in dementia care [16–18], with promising effects on a range of outcomes in other kinds of clinical care [19, 20]. Nonetheless, the effectiveness of person-centred approaches to TB care is yet to be systematically and robustly investigated, especially in LMICs. It remains to be determined which principles and dimensions of PCC could improve TB care outcomes, but for the purposes of this review we focus on the concepts of person narrative, care collaboration and continuity. Person narrative focuses on decision-making based on rich information about the subjective experiences and attitudes of the patient, beyond biomedical symptoms; care collaboration includes the degree of collaboration between caregiver and patient during assessment, decision-making and care planning; and continuity refers to care as an on-going process that, in conjunction with person narrative, can be adapted, revised and reinterpreted [21].

We report a scoping review to inform the development of a person-centred package of TB care in primary healthcare (PHC) clinics in rural South Africa [14]. The objectives of the scoping review are two-fold:

1. To describe the nature and extent of TB and common mental (anxiety, mood) and substance use disorder comorbidity in LMICs and the BRICS region.
2. To explore person-centred care approaches to TB and mental and substance use disorder comorbidities in LMICs and the BRICS region.

## Methods

### Key definitions

There is no universally accepted, unified definition for person-centred care (PCC) [22, 23]. The term is often used interchangeably with related concepts such as “patient participation”, “patient empowerment” and “patient-centredness” [22–26]. Despite conceptual congruence in terms of dimensions such as a holistic perspective on the patient’s illness experience, an enabling collaborative therapeutic alliance between care giver and receiver [23, 24], and values such as empathy and respect [25], PCC has important distinctions. Approaches such as patient-centred care focuses on interactions in terms of visits and contact episodes; disease management; comorbidity as multiple diseases; body systems as distinct; professionally defined coding; and the evolution of disease. PCC, on the other hand, highlights interrelationships over time; illness episodes in a life-course perspective; the interrelated nature of body systems, diseases and morbidity; coding systems that allow for service user concerns; and considers both the evaluation of disease and people’s illness experience [27]. Person-centredness in care aims to understand 1) a person’s subjective experiences and interpretations of illness by considering psychosocial dimensions alongside biomedical symptomology, 2) adhere to the centrality of shared decision-making and parity, and 3) the primacy of relationships in care and treatment [28].

### Study design and literature search strategies

A scoping review was conducted in line with criteria outlined by Arksey and O’Malley [29] and the Johanna Briggs Institute [30]. The following steps were followed: 1) Clear research objectives were developed and refined among research team members, following a brief review of key literature; 2) Relevant studies were identified by members of the research team; 3) Key studies were selected by members of the research team; 4) Data extracted from selected studies were charted; 5) Findings were collated, thematically analysed and reported using Preferred Reporting Items for Systematic reviews and Meta-Analyses Extension for Scoping Reviews (PRISMA-ScR) [31].

### Study selection and data abstraction

The search included English-language studies published within the period 2000–2019 in peer-reviewed and grey literature. From May to October 2018, two researchers (AvR and AD) independently searched PubMed Health Source (Academic/Nursing Edition); MEDLINE; PsychARTICLES; Psychinfo; the World Health Organization website; Google and Google Scholar. Different iterations of search terms where used in combination for each research objective (Table 1). For example, for Objective 1, the terms *tuberculosis OR TB AND mental disorders OR mental illness OR mental health* were entered, with the limiters *Abstract, Publication date from 2000/01/01 to 2018/12/31, English, Adult: 19+ years, Adult: 19–44 years.* For a list of all search terms and limiters employed see Table 1. The reference lists of key publications with high relevance were also searched, and PubMed’s similarity function was used to find articles excluded by the search terms. Two researchers independently reviewed the titles of search results for appropriateness, followed by a review of article abstracts. Where differences between the two researchers were noted, a third researcher was consulted. The inclusion criteria were: evidence from LMICs and BRICS countries, published since year 2000 and, and focused on both TB and mental disorders. The citations of these initial searches were imported into Endnote X9 (bibliography management software) [32] and exported to systematic review application Rayyan [33] for management and review. These systematic steps are presented in PRISMA flowchart format [34]. Once a final list of relevant articles was identified for each research objective, electronic versions were imported into NVivo version 12.1 (a qualitative data analysis software aiding thematic analysis of literature or qualitative research) [35] for management. A thematic analysis of the final group of articles were conducted in order to generate a narrative account of existing literature relevant to the two research objectives [29].

**Table 1:** Complete search strategy by objective and database

Database	Date	Search terms applied	Limiters applied
Objective 1: Comorbidity between tuberculosis and mental disorders			
PubMed	16/05/2018	((((“tuberculosis”) OR TB) AND “mental disorders”) OR mental illness) OR mental health	Abstract, Publication date from 2000/01/01 to 2018/12/31, English, Adult: 19+ years, Adult: 19–44 years.
Ebscohost: Health Source (Academic/Nursing Edition); Medline; PsychARTICLES; Psychinfo	05/07/2018	TI (tuberculosis or tb) AND AB (mental health or mental illness or mental disorder or psychiatric illness)	Abstract, Publication date from 2000/01/01 to 2018/12/31, English, Adult: 19+ years, Adult: 19–44 years.
Objective 2: Person-centred tuberculosis care			
PubMed	23/07/2018	(“patient centred care”) OR “person centred care”) AND “tuberculosis”	Abstract, Publication date from 2000/01/01 to 2018/12/31, English, Adult: 19+ years, Adult: 19–44 years.
PubMed	23/07/2018	(tuberculosis or tb) AND AB (mental health or mental illness or mental disorder or psychiatric illness) AND AB (person centred care or patient centred care or holistic care or relationship centred care or individualised care)	Abstract, Publication date from 2000/01/01 to 2018/12/31, English, Adult: 19+ years, Adult: 19–44 years.
Ebscohost: Health Source (Academic/Nursing Edition); Medline; PsychARTICLES; Psychinfo	23/07/2018	(tuberculosis or tb) AND AB (person centred care or patient centred care or holistic care or relationship centred care or individualised care)	Abstract, Publication date from 2000/01/01 to 2018/12/31, English, Adult: 19+ years, Adult: 19–44 years.
PubMed	14/12/2018	(((“persons”[MeSH Terms] OR “persons”[All Fields] OR “person”[All Fields]) AND centred [All Fields] AND care [All Fields]) OR ((“persons”[MeSH Terms] OR “persons”[All Fields] OR “people”[All Fields]) AND centred [All Fields] AND care [All Fields])) AND (“tuberculosis”[MeSH Terms] OR “tuberculosis”[All Fields]) AND (hasabstract [text] AND (“2000/01/01”[PDAT]: “2018/12/31”[PDAT]) AND (“adult”[MeSH Terms] OR “adult”[MeSH Terms:noexp]))	Abstract, Publication date from 2000/01/01 to 2018/12/31, English, Adult: 19+ years, Adult: 19–44 years.

## Results

**Fig. 1:**
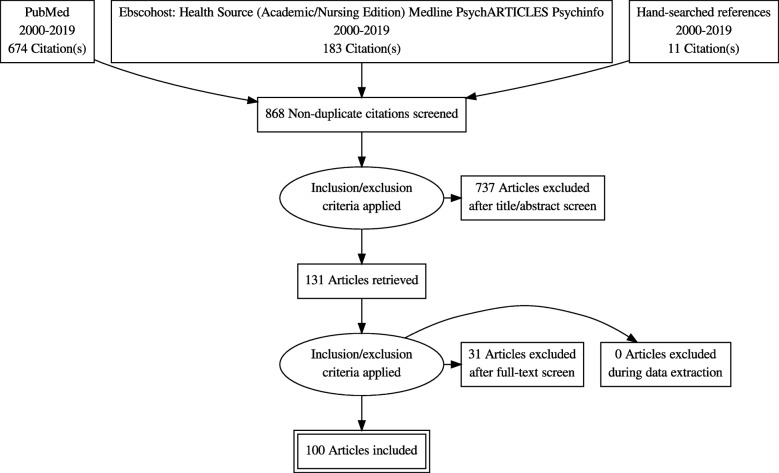
Search results for papers describing the nature and extent of tuberculosis and mental (anxiety, mood and substance use) disorder comorbidity in LMICs and BRICS (2000–2019)

For Objective 1, a total of 868 articles were initially identified (see Fig. 1). After screening the titles and abstracts for appropriateness, 737 articles were excluded because: The scope of the article did not include TB and mental disorders; did not focus on LMICs or BRICS; fell outside the 2000–2019 time limit; did not focus on adults. In the next phase, 131 articles were screened in full, using the same limiters, resulting in the exclusion of 31 articles. Thus 100 articles were included in the thematic analysis.

**Fig. 2:**
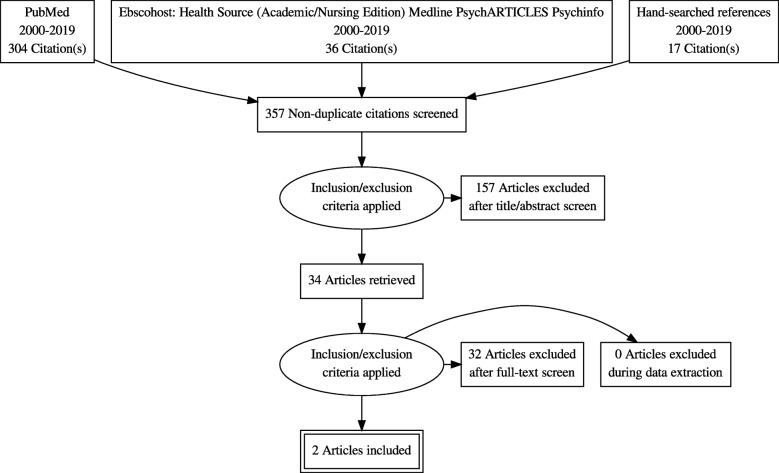
Search results for papers describing person-centred care approaches to tuberculosis and mental disorder comorbidities in LMICs and BRICS (2000–2019)

In terms of Objective 2, an initial number of 357 articles were identified (Fig. 2). After title and abstract screening, 157 articles were excluded because: The scope of the article did not include person-centred/patient-centred/community-centred approaches to TB care; did not focus on LMICs or BRICS; fell outside the 2000–2019 time limit; did not focus on adults. Thirty-two articles were excluded following full-text review, with only two papers included in the thematic analysis.

Several themes emerged during analysis. Four broad TB/mental disorder comorbidities were described in the literature, namely alcohol use and TB, depression and TB, anxiety and TB, and general mental health and TB (meaning that no specific disorders or psychiatric symptoms were identified). Studies generally did not include a clinical psychiatric diagnosis according to international guidelines, instead depending on measures such as quality of life as a proxy for mental wellness, or measurements of mental distress. Therefore, these studies did not strictly focus on TB and mental health comorbidities, but rather on the nexus between TB disease and psychological symptomology. For the different comorbidities, sub-themes that emerged included Comorbid TB and mental illness symptom rates; Associations between TB and mental illness symptoms; TB disease progression and mental illness symptoms; and Factors influencing relationships between TB and mental illness symptoms.

### Comorbid TB and mental illness symptom rates

Rates of mental illness symptoms among TB patients were collated according to depressive symptoms, anxiety, alcohol use, and general mental health (Table 2). Studies that did not specify depression, anxiety or alcohol use – for instance psychological distress – were included under general mental health. Prevalence of depressive symptoms varied dramatically from 9% in Zambia [59] to 84% in India [46]. Likewise anxiety-related symptoms from 2% in India [46] to 47% in Pakistan [43] – gender differences were not widely explored. Alcohol use was measured in a variety of ways: questions on alcohol use habits, references to alcoholism, and use of Alcohol Use Disorders Identification Test (AUDIT). Indications of harmful alcohol use ranged from 4% in Kazakhstan to 67% in Estonia [81]. Men were far more likely to engage in harmful alcohol use.

**Table 2:** Rates of tuberculosis and mental illness symptom comorbidity

Citation	Country	Population group	Comorbidity rates											
			Depression			Anxiety			Alcohol use			General mental health		
			Male	Female	Total	Male	Female	Total	Male	Female	Total	Male	Female	Total
Aamir et al. 2010 [36]	Pakistan	TB patients	–	–	35.3% high depression/anxiety; 36.9% moderate depression/anxiety	–	–	35.3% high depression/anxiety; 36.9% moderate depression/ anxiety	–	–	–	–	–	–
Kendall et al. 2013 [37]	South Africa	MDR-TB patients	–	–	–	–	–	–	–	–	63% recent use	–	–	3% psychiatric comorbidity
Ugarte-Gil et al. 2013 [38]	Peru	TB patients	–	–	37%	–	–	–	–	–	–	–	–	–
Ahmad et al. 2016 [39]	Pakistan	MDR-TB patients	–	–	76% (depression risk)	–	–	–	–	–	–	–	–	–
Xu et al. 2017 [40]	China	TB patients	–	–	–	–	–	–	–	–	–	63.1%v psychological distress	70.3% psychological distress	65.2% psychological distress
Theron et al. 2015 [41]	South Africa	TB patients	–	–	–	–	–	–	–	–	26% regular and heavy use	–	–	22% psychological distress
Tola et al. 2017 [11]	Ethiopia	All types of TB	–	–	–	–	–	–	–	–	15% hazardous and harmful use	–	–	48.9% risk for psychological distress
Laprawat et al. 2017 [42]	Thailand	TB patients	–	–	–	–	–	–	–	–	24.4% hazardous and harmful use	–	–	–
Hussain et al. 2008 [43]	Pakistan	TB patients	–	–	46.3%	–	–	47.2%	–	–	–	–	–	–
Singh et al. 2015 [44]	India	TB patients	–	–	12%	–	–	5%	–	–	4%	–	–	24%
Chandrashekar et al. 2012 [45]	India	TB patients	–	–	–	–	–	–	–	–	–	–	–	46%
Srivastava et al. 2014 [46]	India	TB patients	–	–	84%	–	–	2%	–	–	–	–	–	–
Duko et al. 2015 [47]	Ethiopia	TB patients	–	–	43.4%	–	–	41.5%	–	–	–	–	–	40.6%
Mohammed et al. 2015 [48]	Sudan	TB patients	–	–	–	–	–	–	–	–	–	–	–	13.1% poor mental quality of health
Kehbila et al. 2016 [49]	Cameroon	TB patients	22.6%	38.5%	61.1%	–	–		–	–	–	–	–	–
[50]	Kazakhstan	TB patients	–	–	–	–	–	–	–	–	10.3%	–	–	–
Koyanagi et al. 2017 [4]	48 LMICs	TB patients	–	–	23.7%	–	–	–	–	–	–	–	–	–
Das et al. 2014 [51]	India	MDR-TB HIV co-infected patients	–	–	16%	–	–	–	–	–	–	–	–	–
Ambaw et al. 2017 [52]	Ethiopia	Newly diagnosed TB patients	50.6%	58.1%	54%	–	–	–	–	–	–	–	–	–
Scuffell et al. 2017 [53]	Peru	XDR-TB patients	–	–	10.2%	–	–	6.1%	–	–	20.4% current alcohol use 26.5% Past alcohol use	–	–	22.4% mental disorder
Vega et al. 2004 [54]	Peru	MDR-TB patients	–	–	52.2%	–	–	8.7%	–	–	–	–	–	–
Peltzer et al. 2012 [55]	South Africa	PHC TB patients	–	–	–	–	–	–	–	–	23.3% harmful/hazardous users	33.9%	32.2%	32.9% psychological distress
Dos Santos et al. 2017 [56]	Brazil	Hospitalised TB patients	–	–	31.4%	–	–	38.4%	–	–	–	–	–	–
Galhenage et al. 2016 [57]	Sri Lanka	TB patients	–	–	25.2% (inpatient) 8.5% (clinic)	–	–	12.6% (inpatient) 17.6% (clinic)	–	–	–	–	–	–
Ige & Lasebekan 2011 [58]	Nigeria	TB patients	–	–	45.5%	–	–		–	–	–	–	–	–
Van den Heuvel et al. 2013 [59]	Zambia	TB-HIV co-infected patients	–	–	9.3% major depressive disorder	–	–	7.8% generalised anxiety 27.9% any anxiety	–	–	–	–	–	30.9% current suicidality
Zaridze et al. 2009 [60]	Russia	Adults with heavy alcohol use	–	–	–	–	–	–	2.4%^a^ (TB cause of death)	0.4%^a^ (TB cause of death)	–	–	–	–
Augusto et al. 2013 [61]	Brazil	Adult TB patients	–	–	–	–	–	–	–	–	15%	–	–	–
Deponti et al. 2013 [62]	Brazil	Tertiary emergency department patients	–	–	–	–	–	–	–	–	34.6%	–	–	–
Hermosilla et al. 2017 [63]	Kazakhstan	Adult TB patients	–	–	–	–	–	–	–	–	10.3%	–	–	–
Jankowska-Polanska et al. 2015 [64]	Poland	Hospitalised TB patients	–	–	–	–	–	–	31%	7.9%	–	–	–	–
Lackey et al. 2015 [65]	Peru	Adult TB patients	–	–	–	–	–	–	–	–	18.9%	–	–	–
Louw et al. 2016 [66]	South Africa	PHC TB patients	–	–	–	–	–	–	–	–	26.8%	–	–	–
Louw et al. 2012 [67]	South Africa	PHC TB patients	–	–	–	–	–	–	–	–	23.3%	–	–	–
Méda et al. 2014 [68]	Burkina Faso	PHC TB patients	–	–	–	–	–	–	–	–	30.1%	–	–	–
Miller et al. 2016 [69]	Russia	TB patients with alcohol use disorders	10.6%	25.7%	13.3%	–	–	–	18.8 (AUDIT)	15.8 (AUDIT)	18.3 (AUDIT)	–	–	–
O’Connell et al. 2013 [70]	Zambia	PHC TB patients	–	–	–	–	–	–	32.2%	5.9%	–	–	–	–
Thapa et al. 2014 [71]	India	PHC TB patients	–	–	–	–	–	–	25%	3.7%	20.3%	–	–	–
Naidoo et al. 2013 [72]	South Africa	PHC TB patients	–	–	–	–	–	–	–	–	16.4% (medium risk) 5.6% (high risk)	–	–	25% (severe psychological distress)
Peltzer et al. 2014 [73]	South Africa	PHC TB patients	–	–	–	–	–	–	–	–	26.8%	–	–	83.6% (psychological distress)
Peltzer et al. 2013 [74]	South Africa	PHC TB patients	–	–	–	–	–	–	–	–	23.3%	–	–	81% (psychological distress)
Peltzer et al. 2012 [75]	South Africa	PHC TB patients	–	–	–	–	–	–	31.8%	13.0%	23.2%	–	–	–
Bumburidi et al. 2006 [76]	Kazakhstan	TB patients	–	–	–	–	–	–	–	–	4%	–	–	–
Fleming et al. 2006 [77]	Russia	TB patients	–	–	17% moderate depression 43% mild depression	–	–	–	–	–	62% alcohol abuse/dependence	–	–	–
Krupitsky et al. 2006 [78]	Russia	Patients with substance use disorder	–	–	–	–	–	–	–	–	53% diagnosed with TB	–	–	–
Gelmanova et al. 2007 [79]	Russia	TB patients	–	–	–	–	–	–	–	–	19.9% alcoholism at treatment initiation among non-defaulting MDR-TB patients	–	–	–
Jakubowiak et al. 2007 [80]	Russia	TB patients	–	–	–	–	–	–	–	–	24% abuse alcohol 47% of defaulters abuse alcohol	–	–	–
Kliiman & Altraja 2010 [81]	Estonia	TB patients	–	–	–	–	–	–	–	–	36.9% non-defaulters 67.3% defaulters	–	–	–
Duarte et al. 2009 [82]	Brazil	TB patients	–	–	–	–	–	–	–	–	5.3% alcoholism (mortality risk)	–	–	0.9% mental disorders (mortality risk)
Paulo & Peixoto 2016 [83, 84]	Angola	TB patients	42.5%	57.5%	49.4%	–	–	38.3%	–	–	–	41.2%	58.8%	44.4% psychological distress
De Araujo et al. 2014 [85]	Brazil	TB patients	–	–	–	–	–	–	–	–	–	–	–	38.3% CMD 21.4% depressive /anxious mood 40.9% somatic symptoms 31.2% energy reduction 6.5% depressive thoughts
Ndishimye et al. 2017 [86]	Romania	TB patients	–	–	–	–	–	–	–	–	56% past/current alcohol use	–	–	–
Van den Hof et al. 2013 [87]	Kazakhstan	MDR-TB	–	–	–	–	–	–	–	–	6%	–	–	–
Vijay et al. (2010) [88]	India	New TB patients under DOTS	–	–	–	–	–	–	–	–	49.1% alcoholic	–	–	–
Priedeman et al. 2018 [89]	Ukraine	TB patients	–	–	–	–	–	–	–	–	25.9%	–	–	–
Shin et al. 2010 [90]	Russia	TB patients	–	–	–	–	–	–	70.6% (lifetime alcohol disorder) 33.3% (abuse) 37.2% (disorder)	28.3% (lifetime alcohol disorder) 13% (abuse) 15.2% (disorder)	–	–	–	–
Patel et al. (2016) [91]	India	TB patients	–	–	–	–	–	–	–	–	58%	–	–	–
Finlay et al. 2012 [92]	South Africa	TB patients	–	–	–	–	–	–	–	–	22.9% alcohol use new patients 31.3% alcohol use retreatment patients	–	–	–
Salles et al. 2004 [93]	Brazil	TB patients	–	–	–	–	–	–	–	–	12.9% alcoholism	–	–	–
Suhadev et al. 2011 [94]	India	TB patients	–	–	–	–	–	–	–	–	29% consume alcohol 48% low risk 29% hazardous 7% harmful 16% alcohol dependence	–	–	–
Kolapan et al. 2007 [95]	India	TB patients	–	–	–	–	–	–	–	–	5% alcoholism	–	–	–
Santha et al. 2002 [96]	India	TB patients	–	–	–	–	–	–	–	–	25% alcoholism	–	–	–
Roy et al. 2015 [97]	India	TB patients	–	–	–	–	–	–	–	–	49.4% alcohol use among defaulted TB patients	–	–	–

*AUDIT* Alcohol Use Disorders Identification Test, *CMD* Common mental disorder, *DOTS* Directly observed treatment short course, *MDR-TB* Multi-drug resistant tuberculosis, *PHC* Primary health care, *TB* tuberculosis, *XDR-TB* Extensively drug-resistant tuberculosis

^a^Calculated from presented data

### Associations between TB and mental illness

Several studies explored associations between mental illness symptoms and TB disease. One metanalysis of the period between 2000 and 2014 suggested that as alcohol consumption increases, so does the risk of contracting TB and alcohol-attributable TB mortality. The study estimated that alcohol consumption was responsible for 17% of TB incidence and 15% of TB mortality [98]. The likelihood of TB treatment non-adherence is increased by heavy alcohol use [41, 65, 70, 97]. Alcohol use intersects with TB biologically (through immunosuppression) and socially (increased exposure in closed, poorly ventilated spaces such as taverns and bars) [94, 98]. Further, both alcohol use and TB are “diseases of poverty”, compounding risk in poorer people [94, 99]. TB patients who use alcohol are perceived to be “difficult” by healthcare workers, poorly cooperative and challenging to manage [100]. Patients with TB who also use alcohol were more likely to exhibit risky behaviours and are at a higher risk of contracting HIV [69]. The combination of HIV co-infection and heavy alcohol use prolongs the duration of cough prior to presentation at a health facility [41].

In terms of the influence of the disease on emotional well-being, several studies highlighted impact of a TB diagnosis on mental health. The period following diagnosis is significantly associated with subsyndromal depression, and brief depressive episodes [4]. This emotional toll may be mediated more through role limitation and increased mental distress than physical symptoms [39]. Social stigma and isolation may entrench such feelings [54, 101–104]. A TB diagnosis is associated with a loss of self-esteem, fears of rejection and infecting loved ones [105]. Psychological distress appears to be exacerbated by heavy alcohol use, female gender, previous episodes of TB, and increased morbidity [41]. A Peruvian study reported that people suffering both from TB and HIV expressed a need for support from a psychologist with shortages of mental health professionals impacting negatively on patients’ mental health [103]. These effects are amplified in MDR-TB. A study evaluating the quality of life associated with MDR-TB found that patients suffered from severe physiological, financial and psychological challenges, which persisted beyond treatment completion, and resulted in survival adaptation, poor general health, loss of freedom and agency, and a lack of social participation [102].

### TB disease progression and mental illness symptoms

#### Diagnosis phase and mental illness

The exact causal pathways between TB disease and mental illness are unclear. Few studies have systematically explored longitudinal dimensions between mental illness symptoms and the progression of TB disease, where the diagnosis and treatment initiation stages pose substantial risk for mental illnesses. At diagnosis the following are common: anxiety related symptoms [47, 104], related to fears of death, serious illness, and limited knowledge of TB disease [106, 107], depressive feelings, feelings of worry and embarrassment as well as shame [105]. A TB diagnosis can lead to shock, anxiety and shame, with high levels of psychological distress [55, 103, 107]. At this phase, psychological issues are more severe with family, community and social issues becoming more salient during and after the treatment phase [108]. One study found TB patients with elevated levels of psychological distress were more likely to die during treatment than those with lower levels [41].

Additionally, the risk of mental illness is greater in MDR-TB. MDR-TB patients have a high likelihood of having depression, throughout the illness experience [39]. One study suggested that mental, social and physical challenges posed by MDR-TB were related to deteriorating health-related quality of life scores as the disease progresses [39]. A review of studies on MDR-TB reported that MDR-TB patients face a range of psychological issues, including emotions of hopelessness and fear, accentuated by being conscious of the finality of MDR-TB treatment as a last option [108].

#### Treatment initiation phase and mental illness

TB patients with depressive symptoms are more likely to experience negative TB outcomes than those without such symptoms [38]. Several studies involving mental health measures suggest TB treatment adherence reduces mental illness symptoms. Mental health domains of health-related quality of life assessments have shown to significantly improve following initial phases of TB treatment [39, 101, 109–111]. Risk of developing depression has shown to decrease over the course of TB treatment [112] and adherence shown to improve depressive symptoms of patients suffering from MDR-TB [51].

Initial emotional shock and increased mental health toll, along with lack of health education and emotional support during diagnosis and treatment initiation, elevate this period as critical for emotional and educational intervention [41, 103, 108]. In many countries screening and treating psychological challenges among persons diagnosed with TB is limited. This is especially true for alcohol use disorders and its associated impact on prognosis during treatment; people in TB retreatment programmes are more likely to show risky alcohol use patterns [94]. Further, national TB programmes in countries such as South Africa and India primarily frame TB treatment in biomedical terms, focusing on clinical outcomes such as sputum conversion. However, patients may not regard their treatment as successful due to the impact on the disease and treatment episode – this includes a breakdown in social relationship ties, job loss, social exclusion and a loss of identity [102, 113].

### Factors influencing relationships between TB and mental illness symptoms

#### Factors related to gender

The literature identified several important factors that influence relationships between TB and different dimensions of mental illness. Gender is an especially pronounced factor: during diagnostic and treatment phases of TB treatment, women have consistently shown to have significantly poorer mental health outcomes, including general mental health [39], anxiety [47], depression [4, 49, 52, 59, 84], psychological distress [40, 41, 83, 114], suicidality [59] and mental health-related quality of life. However in one exception, men scored higher than women in an outpatient hospital setting in Uttar Pradesh, India [46]. Regarding alcohol use, men are more likely to consume alcohol at harmful levels prior to and during a TB episode [55, 90, 94, 104, 115]. Men also seem less likely to adhere to treatment regimens [116]. Women may cope differently with TB, employing strategies such as positive stress management, planning and seeking emotional support, whereas men use humour and illicit drugs [104]. A Peruvian study suggested younger men have a pronounced need for emotional support and caregiving from their family – likely from mothers and sisters. The study also highlighted partner support was significantly more important for women [103]. Family support has been shown to be important in the prevalence of depression among TB patients [52, 117]. Women are more likely to attempt alcohol cessation and report negative consequences of drinking on personal relationships, and less likely to jeopardise their personal safety due to alcohol use [90].

#### Factors related to socioeconomic and education background

Patients from lower education backgrounds are more likely to suffer from depressive symptoms [38, 46, 52] and psychological distress [40, 55], and more likely to heavily consume alcohol [94]. Higher education levels on the other hand has been linked to higher mental health-related quality of life [109]. Patients from lower socioeconomic backgrounds are less likely to adhere to treatment [116], more likely to suffer depression [4, 38, 46, 117] and psychological distress [40, 55], and more likely to be heavy alcohol users [94, 115]. HIV/TB coinfection is associated with depressive symptoms [49, 117], while tobacco smoking has also been associated with harmful alcohol use [55] and depression [4] among TB patients. Retreatment, discontinuing treatment [49], and history of TB [41] may make depressive symptoms more likely, while patients who relapse treatment are more likely to be heavy alcohol drinkers [94]. Finally, heavy alcohol use is related to higher levels of depression among TB patients [41], similarly to diabetes [4].

### The paucity of person-centred care

The paucity of person-centred perspectives in LMIC and BRICS approaches to address comorbidities between drug-sensitive and drug-resistant TB on the one hand, and mental disorders on the other is clear from the scoping review results. Focusing on person-centred care (as opposed to patient-centred care) and following the required components of patient narrative, collaboration and continuity [21], our search yielded two studies, both in Nepal, that explicitly aimed to develop more person-centred care to address the psychosocial needs of MDR-TB [118, 119].

Some studies have aimed to develop patient-centred models of care, for instance, a South African intervention that trains social workers to support people suffering from drug-resistant TB [120]. In a study in Nicaragua, TB clubs and home visits were implemented to address TB patients’ internalised stigma, with some success [121]. Medecins Sans Frontieres (MSF) developed and implemented a home-based MDR-TB treatment programme in Northern Uganda, which was found to be acceptable to patients, families, communities and healthcare workers [122]. An Ethiopian study investigated the extent to which the directly observed treatment short course (DOTS) strategy is patient-centred, showing that it falls short of achieving such care [123].

Baral and colleagues [118] aimed to explore challenges people face receiving MDR-TB care in Nepal, and to co-develop a person-centred intervention with patients and families. The research underlined the financial and social strains that conventional MDR-TB care places on individuals, including increased expenditure accessing care, and challenges resulting from removal from social networks, stigmatisation, loneliness and medication side effects. An intervention combining psychosocial counselling and added financial support was developed with positive effects on treatment outcomes. Further research from Nepal aimed to develop and test a psychosocial support package to be integrated within routine MDR-TB care [119]. Stakeholder voice was again prioritised, with patient, family member and service provider perspectives elicited, providing understanding of the physical and psychological impact of MDR-TB; the intersection between health facilities, health information and psychological dimensions of disease; patient contact with family and social networks; personal living arrangements; and financial circumstances and livelihood. Developing the intervention collaboratively assisted in strengthening stakeholder capabilities, opportunities and motivations, resulting in more sustained psychological support throughout treatment, especially for patients with limited social support and who faced stigma [119]. While these developments are promising, little to no empirical evidence exists on person-centred care models for drug-sensitive TB programmes, or the impact or effectiveness of person-centred care in improving individual, clinical and programmatic outcomes.

## Discussion

This review aimed to explore the nature and extent of comorbidities between TB and mental illness symptoms in LMICs, and to identify and describe person-centred approaches to address such comorbidities. As expected, studies that focused on intersections between TB and mental illness symptoms in LMICs varied considerably according to scope, size, study design and robustness, making comparisons difficult. However, our findings identified four broad tuberculosis and mental disorders comorbidities namely alcohol use and tuberculosis, depression and tuberculosis, anxiety and tuberculosis, and general mental health and tuberculosis. Despite the wide variance between research in different countries, it is clear that experience of a TB disease episode is often accompanied by or related to CMD and alcohol use symptoms (see a summary of main findings in Table 3).

**Table 3:** Key findings on the comorbidity of tuberculosis and mental illness

• High co-morbidity of mental illness with TB (name the mental illnesses) and very pronounced with MDR	
• Where relationships between mental illness and TB have been shown, we see consistent patterns to those for people without TB: women tend to suffer more depression; men more alcohol; socioeconomic and less education also more associated with more mental illness.	
• Relationships bidirectional (most clearly for alcohol use disorders)	
• Mental health needs are most pressing during the diagnostic and treatment initiation phases of TB treatment	
• Some evidence of resolution of mental illness symptoms with TB treatment, but also concern that for MDR-TB symptoms persist for long periods and even after treatment	
• Patterns between TB and mental illness with poverty similar. Poverty associated with higher incidence of both conditions and TB also perpetuates poverty. The combination of poverty, TB and alcohol use disorders appears particularly severe.	

In contrast to research on HIV, there is limited empirical data globally on the prevalence of mental illness symptoms among people suffering from TB [59]. Nonetheless, mental illness rates are higher among people with physical illness compared to the general population, and it might even be higher for people with TB – depression and anxiety certainly seems to be higher [2]. Results from the World Health Survey, conducted among 48 LMICs, suggest comorbid TB and depression significantly worsen health outcomes across all domains, exceeding burdens of TB and depression in isolation. Further, people suffering from TB are almost at a fourfold increased risk of experiencing depressive episodes [4]. Our review demonstrated that a body of evidence is growing in LMICs, rendering an urgent need for standardised concepts and measurements that incorporate person-centred systems of care.

Our findings suggest that initial diagnosis and treatment phases of the TB episode are particularly salient in preventing and treating mental health challenges, as baseline mental illness symptoms negatively affect the treatment phase [38]. Treating mental health challenges is a clinical priority, with psychological distress potentially doubling the risk of non-adherence [41]. Indeed, comorbid mental illness – including substance use – is a major consideration in treatment non-adherence which, in addition to the impact on individuals, has wider public health implications, including significant contributions to transmission and emergence of drug-resistant disease [2].

Research on TB and mental illness comorbidities that stretch beyond describing cross-sectional prevalence rates are rare, with very little attention paid to longitudinal dimensions of the trajectory of comorbidity [3]. Causal pathways between TB and depression are both complex and multidirectional, manifesting in biological, pharmaceutical and psychosocial mechanisms, though the extent to which these pathways contribute to comorbidity burden remains unclear [3, 38]. One mechanism suggests that depression negatively affects self-care in TB treatment, thereby increasing chances of treatment non-adherence and failure [4]. Further, alcohol use before and during treatment has a strong negative effect on a myriad of outcomes, manifested in complex, bidirectional causal chains. Poverty can lead to increased alcohol use, which in turn may lead to an increased likelihood of contracting TB, not being diagnosed in a timely manner, and not adhering to treatment; also, alcohol use may lead to downward social mobility, again creating social conditions favourable for TB disease progression [99]. The review illustrated that there are key nuances in TB and mental illness comorbidity; women are more prone to depression, while men are more likely to engage in harmful alcohol use. Men are also less likely to adhere to treatment regimens. The gendered dimension of comorbidity is an important consideration in TB programme design and again underlines the need for person-centredness. Other vulnerabilities such as poverty, lower levels of education and older age groups present further opportunities for more targeted interventions.

Health system barriers – such as fragmented, non-dependable health information systems, limited patient management, referral and communication fractures between levels of care – further compound challenges during the TB disease episode [124]. These are unfortunately features of many national health systems in LMICs, creating a troubling context for reforms towards person-centredness [125]. Importantly, many challenges faced by TB patients lie outside the formal health system sphere – transport problems, medication reactions and food assistance [126]. A particular silence in current literature relates to occupational dimensions of TB, both prevention and treatment and care. The hidden epidemic of TB among healthcare workers, and the resultant care approaches by employers require urgent attention.

Despite recent growth in person-centred discourse in global health (for instance in the SDGs, Lancet Global Health Commission on High-Quality Health Systems in the SDG Era, WHO Framework for People-centred Health Systems), our review showed that little to no person-centric TB treatment approaches are being deployed in LMICs, with the exception of the two studies in Nepal, which focused on psychosocial support for people suffering from MDR-TB [118, 119]. While there is an increase in attention to patient-centredness in TB care which include patients and their families in decision-making, person-centredness considers the whole person in a wider context rather than a focus on a person’s role as a patient. Very few studies have presented person-centred models of care, as well as comparable empirical investigations into cost effectiveness and programme effectiveness [127]. The person-centred dimensions of patient narrative, collaboration and continuity [21] still fall short in LMIC and BRICS research. Stakeholder voice is especially critical; TB interventions will only be able to achieve positive outcomes with the full inclusion of patients, their families and wider network in the planning and organisation of care [128].

## Table 3: Main findings

This review suggested that there is increasing attention being paid to the intersections of mental illness symptoms and TB disease in LMICs. There are well-known vulnerabilities during a TB episode – including those tied to the patient, their family and broader context, as well as persisting health system ills. At the very least, TB programmes should be targeted enough to be able to address key challenges, especially dimensions of gender, education and socioeconomic background. This review highlighted a common feature of international research comparisons, namely the lack of standardisation and fragmentation in the use of different concepts. The study of mental illness and TB crucially requires a globally accepted framework that details the range and nature of comorbidities, as well as robust methods of measurements. Similarly, there is an identified need for a conceptual framework that sets out the definitions of patient -and person-centred care as it applies to TB. This will require wide consensus on the meaning of these terms and how they are operationalised, while inappropriate terms such as ‘TB suspect’ and ‘treatment defaulter’ – with its subtle judgements and disempowerment – should be replaced by person-centred terminology such as ‘people with presumptive TB’ and ‘person lost to follow-up’ [127].

Several concepts used during this review are not clear cut, and its vagueness in interchangeable use renders searching and comparison difficult. Mental illness symptoms are notoriously difficult to pin down, and many nuances go astray during its operationalisation in research. For example, the degree to which alcohol use become a feature of mental illness is not always made entirely clear by researchers, and the distinction between the initial shocks of a TB diagnosis should be differentiated from formally diagnosed anxiety. Similarly, it is possible that some papers might have been missed during the search due to treatment approaches and care not explicitly being named “person-centred”, “patient-centred” or “people-centred”, rather implicitly inferring dimensions of these terms (for instance, “including the patient in decision-making”.

## Conclusions

The literature clearly showed that the presentation of mental illness – including substance use, anxiety and depression – during the TB disease trajectory, have a range of negative outcomes. This is often exacerbated in LMICs where the majority of people suffering from TB are often faced with complex challenges such as poverty, gender disparities, limited education and failing health systems. Moreover, mental illness and TB comorbidities were found to be more prevalent within the diagnostic phase and later the treatment stages. These phases were found to be associated with high levels of psychological distress thus often requiring more person-centred TB care models. Addressing psychosocial comorbidities with TB in LMICs and BRICS with person-centred TB care in routine care platforms may yield promising clinical and psychosocial outcomes.

## Data Availability

Data sharing is not applicable to this article as no datasets were generated or analysed during the current study.

## Abbreviations

ASSET: heAlth System StrEngThening in sub-Saharan Africa
BRICS: Brazil, Russia, India, China, South Africa
CMDs: Common mental disorders
HIV: Human immunodeficiency virus
LMICs: Low-to-middle income countries
MDR-TB: Multidrug resistant tuberculosis
MSF: Médecins Sans Frontieres
NIHR: National Institute for Health Research
PCC: Person-centred care
SDGs: Sustainable Development Goals
TB: Tuberculosis
WHO: World Health Organization
